# The Geshiyaro Project: a study protocol for developing a scalable model of interventions for moving towards the interruption of the transmission of soil-transmitted helminths and schistosome infections in the Wolaita zone of Ethiopia

**DOI:** 10.1186/s13071-019-3757-4

**Published:** 2019-10-29

**Authors:** Kalkidan Mekete, Alison Ower, Julia Dunn, Heven Sime, Gemechu Tadesse, Ebba Abate, Nebiyu Nigussu, Fikreselasie Seife, Emily McNaughton, Roy Malcolm Anderson, Anna Elizabeth Phillips

**Affiliations:** 1 Ethiopian Public Health Institute, Addis Ababa, Ethiopia; 2 London Centre for Neglected Tropical Disease Research, Department of Infectious Diseases Epidemiology, School of Public Health, Faculty of Medicine, Imperial College London, London, W2 1PG UK; 3 Federal Ministry of Heath, Addis Ababa, Ethiopia

**Keywords:** Schistosomiasis, Soil-transmitted helminths, Elimination, Transmission break, Ethiopia, Water, Sanitation and Hygiene (WaSH), Behaviour Change Communication (BCC)

## Abstract

**Background:**

National deworming programmes rely almost exclusively on mass drug administration (MDA) to children to control morbidity caused by these parasitic infections. The provision of other interventions, consisting of preventive chemotherapy at high population level coverage together with water, sanitation and hygiene (WaSH) and changes in risk behaviour, should enable sustainable control of soil-transmitted helminths (STH) and schistosomiasis and ultimately interrupt transmission.

**Methods/Design:**

Two interventions will be implemented by the project: (i) community-wide biannual albendazole and annual praziquantel treatment with a target of 80–90% treatment coverage (“expanded MDA”); and (ii) provision of WaSH with behaviour change communication (BCC), within the Wolaita zone, Ethiopia. The project has three study arms: (i) expanded community-wide MDA, WaSH and BCC; (ii) expanded community-wide MDA only; and (iii) annual school-based MDA (the current National STH/schistosomiasis Control Programme). The impact of these interventions will be evaluated through prevalence mapping at baseline and endline (after four rounds of MDA), combined with annual longitudinal parasitological surveillance in defined cohorts of people to monitor trends in prevalence and reinfection throughout the project. Treatment coverage and individual compliance to treatment will be monitored by employing fingerprint biometric technology and barcoded identification cards at treatment. WaSH utilisation will be evaluated through school and household level observations and annual WaSH assessment survey. Complementary qualitative surveys will explore practices, cultural and social drivers of risk behaviours, uptake of WaSH and treatment, and assessing the impact of the BCC.

**Discussion:**

The study has the potential to define an ‘End Game’ for STH and schistosomiasis programmes through provision of multiple interventions. Interrupting transmission of these infections would eliminate the need for long-term repeated MDA, lead to sustained health improvements in children and adults, thereby allowing health systems to focus on other disease control priorities.

## Background

Neglected tropical diseases (NTDs) are a group of infectious diseases that affect over 1.4 billion people globally [1]. Helminth infections caused by soil-transmitted helminths (STH) and schistosomes are among the most prevalent NTDs affecting humans who live in poverty [2–4]. Whilst these diseases do not typically cause high mortality, repeated infection from an early age can cause chronic morbidity including malnutrition, growth impairment, and hinder economic development in endemic regions [5–7]. Emphasis in Africa is placed on the four most common STH infections (*Ascaris lumbricoides*, *Trichuris trichiura*, *Necator americanus* and *Ancylostoma duodenale*) and two most common schistosomes (*Schistosoma mansoni* and *S. haematobium*), as these account for most of the helminth disease burden.

The most marked epidemiological attributes of human helminth infections are their highly aggregated distributions so that few individuals harbour a disproportionate number of worms, predisposition of certain individuals to infection, rapid reinfection after treatment with no evidence of strong acquired immunity, and age-intensity profiles that typically show highest infections in school-aged children (SAC) [8, 9]. Hookworm is an exception, since the intensity of infection typically increases with age to plateau in adulthood [10].

Within the past decade, through the direction of the World Health Organisation (WHO), significant progress has been made in implementing large-scale drug treatment programmes targeting STH and schistosomiasis typically through school-based drug administration. The SAC-targeted treatment approach was formulated by the WHO two decades ago when focus was given to age groups with the highest prevalence of STH and schistosomiasis infection and the greatest morbidity burden. Whilst SAC-targeted preventive chemotherapy (PCT) has resulted in a large decline in STH (especially when combined with community-wide treatment to control lymphatic filariasis) and schistosomiasis prevalence and morbidity, recent research suggests that transmission of these parasites cannot be interrupted without expanding treatment to all age groups except in low transmission settings [11–13]. In the past, a key bottleneck to scaling up implementation of PCT to the whole community has been limited access to drugs. In 2012, however, pharmaceutical companies including GlaxoSmithKline and Johnson & Johnson pledged to donate albendazole (ALB) and mebendazole until 2020 to countries with endemic infection to treat SAC most at risk for STH infection [14]. Merck KGaA also committed to donate 250 million tablets of praziquantel (PZQ) per year until the morbidity due to schistosome infection is eliminated [16]. The donation of anthelminthic drugs has allowed countries to scale-up control efforts and some have even started to move beyond SAC either to include adults in mass drug administration (MDA) programmes [15, 16]. Mathematical models of transmission and control suggest that community-based deworming, which includes treatment of adults, can greatly decrease both the prevalence and intensity of infection, with the most pronounced effects in regions where hookworm is the dominant STH species [11, 13–17].

While PCT can greatly reduce morbidity from helminth infection, reinfection typically occurs rapidly after treatment due to the absence of effective acquired immunity [18]. The time scale of such ‘bounce back’ post cessation of MDA is typically fast for STH (1–2 years) and slower for schistosome infections (3–5 years) due to differences in adult worm life expectancies (the longer the adult worm life expectancy the slower the bounce back) [19].

Provision of safe water, sanitation and hygiene (WaSH) is one of the five key interventions within the global NTD roadmap leading to long-term control and eventual elimination [20]. In the USA, South Korea and Japan, for example, where WaSH improvements acted in conjunction with deworming, success was achieved in eliminating STH as a public health problem [21, 22]. This supports the need for an integrated control paradigm. WaSH interventions are diverse, including improvements in water access (e.g. water quality, quantity and distance to water), sanitation (e.g. access to improved latrines, latrine maintenance and waste management), and hygiene practices (e.g. handwashing before eating and/or after defecation, water treatment, soap use, wearing shoes and water storage practices) [12, 23–32]. Empirical evidence linking the benefits of WaSH on concomitant reductions in STH and schistosomiasis is limited and an improved evidence-base with better quantification of the WaSH interventions introduced and the costs entailed may lead to better coordination between the NTD and WaSH sectors [24–26].

To fill this gap, the central aim of the Geshiyaro Project is to provide an evidence-base for a scalable model of interventions for the interruption of STH and schistosomiasis, leading to the cessation of MDA. The Geshiyaro Project will be implemented in Wolaita zone in Ethiopia and will investigate the impact of two sets of interventions: anthelmintic PCT (implementing more frequently and/or extended treatment to the entire population rather than the current school-based programme) and provision of safe water and sanitation alongside behaviour change communication (BCC).

The specific aims are to (i) demonstrate whether planned interventions to control STH and schistosomiasis (MDA, WaSH, BCC) can feasibly break transmission (< 2% community prevalence by quantitative polymerase chain reaction (qPCR)) in a focal geographical area of Ethiopia; (ii) identify effective interventions with an understanding of the associated costs, motivators and barriers associated with each strategy; (iii) assess the feasibility of the different interventions as large-scale control efforts of STH and schistosomiasis, including costs, appropriate use of diagnostics and lessons learned.

The specific objectives are to (i) quantify overall achieved reductions in infection prevalence and average intensity stratified by age (from pre-SAC to adult) within and between the study arms from baseline to endline as measured by Kato–Katz, POC-CCA, haemastix, urine filtration and qPCR; (ii) determine yearly patterns of intervention impact through annual changes in prevalence and intensity of infection in longitudinal sentinel sites within and between the study arms by standard diagnostics (Kato–Katz, POC-CCA, haemastix and urine filtration) across all age groups; (iii) evaluate levels of treatment coverage using fingerprint biometric technology (hereafter biometrics), study ID cards, as well as WHO independent coverage surveys, and the effect on transmission; (iv) identify serial non-complying groups of the population who do not regularly partake in MDA through use of the participants fingerprint; (v) assess the annual change in knowledge on schistosomiasis and STH, and the impact of subsequent behaviour change on transmission; (vi) evaluate the change in access to WaSH, and the impact on transmission; (vii) test and evaluate the more sensitive diagnostic methods of qPCR for parasite DNA detection in stool for their sensitivity, specificity and feasibility for elimination; (viii) develop and validate mathematical models for the prediction of STH and schistosomiasis prevalence after MDA and/or WaSH interventions.

## Methods/design

### Study design

**Fig. 1:**
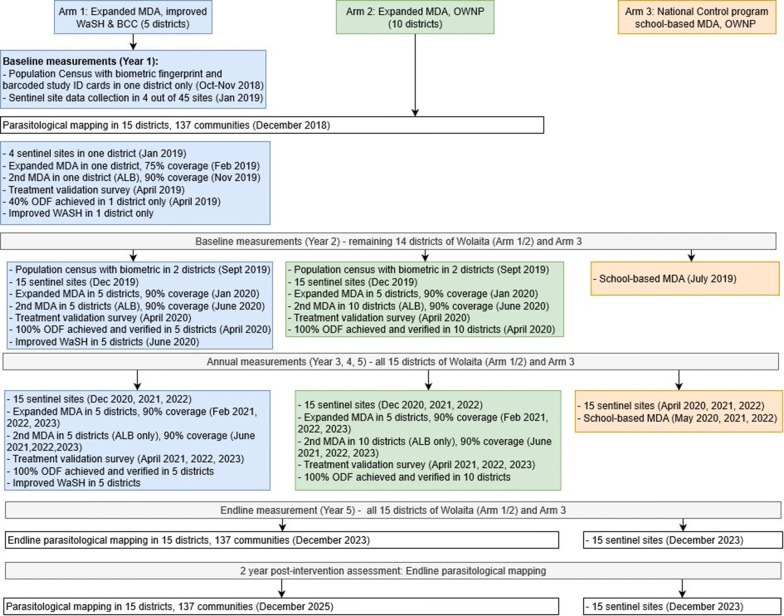
Study design of the three Geshiyaro intervention arms

The Geshiyaro Project has three intervention arms (Fig. 1). The three arms are designed to test which combination of interventions are most effective at reducing STH and schistosomiasis prevalence to a level where transmission may be interrupted. The three arms are:

(i) *Expanded MDA, improved WaSH and BCC in five districts (Wolaita Zone).* “Expanded MDA” will be conducted at several levels. First, PCT will be extended to all the community, including adults and pre-SAC in the MDA eligible population (ALB ≥ 1 year and PZQ ≥ 5 years). Secondly, MDA coverage will be scaled up to reach 90% verified coverage, with 10% confidence interval, in all target age groups. “Improved WaSH” will incorporate clean water provision, sanitation and hygiene. Increased access to clean water supply will be achieved through building both deep and shallow water wells, as well as public taps. Sanitation improvement will be addressed through mobilisation of people to build and use latrines, with a reduction of open defecation through community led total sanitation (CLTS). All WaSH interventions are led by World Vision Ethiopia (WVE). Health education will be aimed at encouraging healthy behaviours and reducing contamination of soil and water by promoting the use of latrines and hygienic behaviour through BCC. This is also led by WVE.
(ii) *Expanded MDA and “One WaSH National Programme” (OWNP): ten districts (Wolaita Zone).* Community-wide deworming of everyone in ten districts (over the age of one-year-old) with ALB (biannual) and PZQ (annual). This will be coupled with the Ethiopian government’s “One WaSH National Programme”. Since 2013, Ethiopia has established a WaSH sector-wide approach under the umbrella of the OWNP, which brings together ministries, development partners, and civil society organisations to a common goal of one plan, one report and one budget. The aims of OWNP are to harmonise and align approaches to WaSH improvement, ensure equity in WaSH provision, and avoid varying financial and procurement procedures by donors.
(iii) *Annual school-based MDA and OWNP (Control): outside of Wolaita zone.* Standard annual school-based deworming of pre-SAC and SAC (2–14 years-old), with ALB, will be maintained through the government nutritional programme of under-fives and the National STH/schistosomiasis Control Programme, respectively. Annual treatment of SAC with PZQ will be carried out in the STH and schistosomiasis school-based treatment programme. Again, this will be combined with the government’s “One WaSH National Programme”.

The project will last seven years and consists of three phases. Phase 1 (2018/2019) is the pilot year in which Arm 1 study activities will take place in one district, Bolosso Sore. Phase 2 (2019–2023) will scale-up activities to all 15 districts of the Wolaita Zone. Phase 3 is two years of observation (2024/2025) where, providing transmission has been interrupted in the community, the regular national control programme MDA will continue in Arms 1, 2 and 3 as per the National Control Programme guidelines. At the end of Phase 3 progress in achieving the final aims and objectives of the project will be evaluated.

### Study setting

The study will be conducted in the Wolaita zone (population 1.9 million), in the SNNPR region of south-western Ethiopia. The Wolaita zone consists of 15 districts or ‘woredas’. A woreda is the smallest functional unit of the health care system covering a population of at least 100,000. Ethiopia conducted a STH and schistosomiasis nationwide mapping survey in all regions of the country between 2013 and 2015 [33, 34]. The surveys showed that both STH and *Schistosoma mansoni* are endemic in Wolaita, with hookworm as the dominant infection in most districts. *Ascaris lumbricodes* and *Trichuris trichiura* are both present in Wolaita at low prevalence. The national deworming programme has been active in the zone since 2013, treating SAC annually, apart from one district which is treated biannually, as per the WHO treatment guidelines. There is thought to be negligible presence of *S. haematobium*.

### Interventions

#### Mass drug administration

All communities in Wolaita (Arms 1 and 2) will receive expanded MDA organised and overseen by the Federal Ministry of Health (FMoH) and implemented at community-level by the Health Extension Workers (HEWs). All community members ≥ 1 year of age will receive ALB against STH, while those ≥ 5 years of age will receive PZQ for schistosomiasis infections [35]. All communities, regardless of arm and prevalence, will receive biannual ALB treatment, and annual PZQ treatment, both with a target of 90% verified coverage, with 10% confidence interval in all age groups. As well as administering ALB and PZQ, HEWs will be trained to collect digital data on individual compliance to treatment. For each treatment record, individuals who present for MDA will be identified using their fingerprint information and, if their fingerprint is not identified, or if they do not consent to their fingerprint being registered, their barcoded study ID card will be used. If the individual does not have an ID card (due to not being reached by census or having lost it) they will have their demographics recorded (e.g. age and sex), fingerprints registered (if they consent) and be given a new study ID card. The HEW will then record if the individual ingests the medication (ALB and PZQ separately) and, if not, their reasons for refusal. At the end of each round of MDA, a list of people registered in census who were not identified during MDA will be fed back to the HEWs for MDA mop-up.

Arm 3 will continue to receive annual school-based deworming through the National Programme in accordance with the WHO guidelines for STH and schistosomiasis control. All deworming (community-wide and school-based) will be administered by HEWs who are responsible for all community-level health interventions, including deworming, immunisations and nutrition support.

#### Water, sanitation and hygiene (WaSH)

In Arm 1, improved WaSH interventions targeting risk behaviour and barriers to access will be implemented through WVE. In Year 1, the aim is for 40% of the communities to be declared open defecation free (ODF) through CLTS, whereby communities work together to build household level, institutional, and community improved pit latrines. CLTS is an approach to addressing open defecation that triggers emotions such as shame and disgust to generate a collective demand for sanitation within a community. In Ethiopia, evidence has shown that HEW-facilitated CLTS can be effective and is currently the strategy promoted by the national OneWASH programme [36]. In addition, WaSH business centres will be established to offer products for purchase (latrine slabs, handwashing stations, soap and brooms) and services (pit digging, super structure labour, and maintenance of infrastructure) through sale and loans. Water infrastructure will be provided to 70% of the population through wells and taps. This will be scaled up to 85% from Year 2. The goal for sanitation is the achievement of 82% basic improved latrines.

#### Behaviour change communication (BCC)

BCC materials will include counselling cards for the HEW and posters, both of which will be designed to promote good hygiene practices to minimise risk of acquiring STH and schistosomiasis infection. Specifically, STH targeted messages will include (i) handwashing with water and soap; (ii) latrine use and reduction of open defecation; (iii) proper household waste management including disposal of infant faeces; (iii) practising safe household water handling, storage and treatment; and (iv) shoe wearing. Messages to reduce acquiring and transmitting schistosomiasis infection will comprise of (i) safe water storage; (ii) avoiding freshwater contact either through bathing or washing clothes; (iii) latrine use as above. In addition, messages to increase participation in the MDA will be delivered, including biometrics and study ID card registration in census districts.

A sales catalogue for the WaSH business centre will also be published advertising products, services and associated costs (including loans).

### Study outcomes

Prevalence and intensity of both STH and schistosomiasis will be evaluated cross-sectionally through baseline, endline 1 (end of Year 5 after four years of study activities) and endline 2 (end of year 7) mapping. In addition, longitudinal sentinel site monitoring of prevalence and intensity of infection in defined cohorts with a sample of 150 individuals stratified by age (pre-SAC, 1–4 years; SAC, 5–14 years; 15–20 years; 21–35 years; 36+ years) will be conducted in each arm from Year 1 to Year 5. The primary outcome of the project is that transmission interruption is achieved as a community-level prevalence ≤ 2% of schistosomiasis and the dominant STH species (primary outcome) and any detectable STH species (secondary outcome) at the end of Phase 2, as determined by qPCR. If prevalence has been reduced to an acceptable level (< 2% by any detectable STH) in a defined primary health care unit (a group of communities served by one health post and HEW), two years of observation will begin where the study population received the national control programme (school-based) MDA. Another assessment will take place at the end of Year 7 (end of Phase 3), two years after the cessation of study activities, to evaluate whether STH/schistosomiasis prevalence has remained low or “bounced back” in the intervening years. Transmission models suggest that reaching a 2% prevalence of STH (any species, diagnosed by qPCR), 24 months after stopping MDA reliably predicts transmission interruption with a positive predictive value of > 90% [37, 38]. This definition will be re-evaluated at endline mapping based on the prevailing infection intensities in the region.

### Sample size and selection of sites

#### Parasitological mapping at baseline and endline

Within the context of elimination, the current WHO guidelines for mapping both STH and schistosomiasis recommend sampling approximately five schools per “health district” [39], which is not suitable given the focality and significant differences in infection between communities within a small area. A previous study on schistosomiasis assessed how many sampling sites and individuals maximised survey accuracy and cost-efficiency [40]. The results showed that district prevalence estimates converged on true prevalence as the number of schools surveyed increased, as expected, though with diminishing returns beyond 10 schools per district. Furthermore, increasing the number of children tested per school led to very small improvements.

Based on these calculations, baseline Geshiyaro mapping survey took place in 137 kebeles (approximately 40% within Wolaita) in December 2018 with a total of 100 individuals sampled per community stratified for equal sample size (20 individuals) by age grouping (pre-SAC (1–4 years), SAC (5–14 years), 15–20 years, 21–35 years, 36+ years) and sex. The overall prevalence across Wolaita showed the prevalence of STH at 11.3% and schistosomiasis at 0.27% by Kato–Katz. Between 26% and 75% of kebeles will be surveyed within each Woreda, with a higher proportion of kebeles being surveyed in small woredas. Communities were randomly selected for sampling. A sample size of 137 communities is sufficient to detect, in children and adults separately, a true reduction in prevalence from 15% at baseline to 0% at the end of the project.

The aims of the mapping are to:

(i) Provide robust estimates of prevalence and intensity of infection within each woreda, and the degree of parasite aggregation within the population and age groupings within each setting; and
(ii) Identify 30 communities within Wolaita (approximately 15% of those surveyed) to purposively select for more intensive impact monitoring, plus compliance to treatment, through sentinel sites.

#### Annual longitudinal sentinel sites (including midline expansion)

The sentinel site longitudinal cohort allows for annual monitoring of a subsample of the population and will explore the association between infection and treatment coverage/compliance (longitudinal drug take up in individuals at each round of treatment), and between infection and access to WaSH facilities. These sites are crucial to understanding impact of compliance to treatment, data obtained from individual barcoded ID cards or fingerprints scanned at MDA, on parasitology (changes in intensity of infection post-treatment). The Geshiyaro Project defines a protocol to sample 150 individuals per site, with 45 sites in total (15 in each arm). Arm 3 will be evaluated in 15 sentinel communities within Southern Nations and Nationalities People’s Region (SNNPR), which are currently being assessed longitudinally through the national monitoring and evaluation sentinel site programme. This figure was determined to allow for anticipated drop out of 10% from the cohort sample. Sample size calculations suggest that, with 95% significance and 80% power, and assuming an intra-class correlation of 0.05, will allow a detection of change in prevalence of at least 10% from an initial prevalence of 11.3%. The study design effect is estimated at 2.5 and is accounted for in the sample size estimation. This sample size equates to one site per 67,000 individuals in the intervention area, which is substantially higher than the WHO recommendation of one site per every 200,000–300,000 individuals. In addition, the design includes monitoring of not just school-aged children, but a broader range of age classes including adults and pre-SAC. Consequently, 30 communities in Wolaita and 15 communities from Arm 3 sites outside of Wolaita were chosen as sentinel sites. An age- and sex-stratified random sample of 150 individuals will be followed in each community selected, totalling 6750 individuals.

Following baseline mapping conducted in December 2018, a list of all communities was categorised as low, medium or high prevalence for both STH and schistosomiasis based on the current WHO guidelines. Each community was then stratified by co-endemicity whereby the number of sites for schistosome infection in each STH category was tabulated. The sentinel sites were randomly selected from each category to ensure a balance of sites across the different infection categories. Due to the focal distribution of schistosomiasis, the ability to capture accurate prevalence data in a defined region will be more sensitive to the number and location of sites compared, for example, to *A. lumbricoides*, *T. trichiura* and hookworm, which tend to be less focal in the magnitude of the prevalence of infection. Additionally, the ability to detect true prevalence is more sensitive both to the diagnostic used and to the number of sites, than to the number of individuals sampled per site [35]. At midline, an additional 15 additional sites in both Arm 1 and Arm 2 will be randomly selected, stratified by baseline prevalence, for parasitological cross-sectional (by age and gender) surveys.

### Data collection

#### Demography (census)

A population census will take place for the Geshiyaro Project prior to any intervention in a total five districts in Wolaita. Three will take place in districts within Arm 1 (Bolosso Sore, Bolosso Bombe and Damot Gale) and two in districts assigned to Arm 2 (Abala Abaye and Damot Weydie). The purpose of the census is to determine an accurate denominator for the MDA using fingerprints and study ID cards, identify (serial) non-compliers to the MDA, and assess baseline sociodemographic and WaSH coverage prior to the start of the intervention. The Ethiopian government is planning to conduct a full census throughout SNNPR in the late part of 2019, which will be used as a denominator for the remaining districts. Between October and December 2018, a pilot census was carried out in one district (Bolosso Sore) to test acceptance of communities to census activities, specifically the fingerprinting technology. The remaining four districts will carry out their census between October and December 2019.

Door-to-door visits will be conducted by enumerators selected by the zonal health bureau and accompanied by a local guide, who is selected by each village, to enrol (consenting) households who will provide the name, age, sex, and relationship to the household head for all individuals residing within the household. Questions regarding household possessions and access to electricity, in addition to the materials used for the household structures (walls, roofing and floors) will be asked to obtain a measure of socioeconomic status. Household surveys to determine access to water and sanitation services (including latrine infrastructure, distance to drinking water point, and water sources for drinking, washing and bathing) will be supplemented by community-wide sanitary surveys. Observational data will be collected on the type of toilet, household structure materials and shoe wearing will be recorded. The GPS coordinates of each household will be recorded.

All individuals within consenting households will be registered with a pre-printed barcoded Geshiyaro study ID card and asked to provide a biometric fingerprint. The accuracy of how many fingers need to be scanned was assessed in the pilot using one, two and four fingers. The result of the pilot analysis showed that taking four fingers yielded the highest accuracy. The barcoded study ID card was not only an additional method of identification, but also to mitigate the limitation of the biometric technology in accurately identifying pre-SAC. For any individuals from the household that were not present, the data collectors will return at another time (in the evening or weekend) to ask for a fingerprint, with a limit of re-visiting an unsuccessful household three times. All individuals, regardless of whether they consented to fingerprint registration or not, will be given a study identification card with a unique study QR code. Every household will also have a unique household study QR code which will be on the back of the household head’s study ID card. The biometrics and study ID cards will be used in all data collection rounds for anonymous data linkage. At each school and health facility within the zone, a WaSH survey will be performed. The total number of students enrolled in each school, and the catchment population and number of households served by each health facility will be documented.

The Geshiyaro Project will use fingerprinting technology to link individuals over multiple data collection activities. Simprints, a technology company, will provide the biometric fingerprint identification system used in this project. Fingerprint characteristics are unique to each individual and will be used therefore to identify individuals reducing the risk of identification errors, such as ID cards being swapped or lost, which can lead to data being linked to the incorrect individual. However, the use of biometric fingerprinting comes with challenges of its own including its reliability in accurately identifying children under the age of five, the number of fingers captured in order to balance data collection burdens with maximizing accuracy, acceptability among community members, and feasibility of using the technology at scale.

#### Parasitology (mapping and sentinel sites)

Parasitological data collection will take place in randomly selected communities at baseline (Year 1), midline (Year 3), endline 1 (Year 5) and endline 2 (Year 7). There will also be annual parasitological data collection in the assigned longitudinal sentinel sites.

Recruitment for the parasitological mapping and sentinel sites will be through door-to-door and school visits with the aid of the HEWs. Individual (≥ 16 years-old) or parental consent (< 16 years-old) for study participation will be obtained. All individuals approached to be registered in sentinel sites will be asked about any plans to move household over the study years.

Duplicate Kato–Katz slides will be prepared from a single (mapping) or two stools over two days (sentinel sites) from all individuals and the number of parasite eggs found in each slide recorded. A single urine sample from each individual will be tested using a POC-CCA test and haemastix. If the latter is positive for blood in urine, then two filtrations on 10 ml of urine will be carried out for each individual and microscopically examined; the number of eggs found in each slide and the corresponding volume of urine filtered will then be recorded. A subset of samples from the sentinel sites in the first year will be stored for subsequent qPCR analysis. At endline all mapping site samples will be analysed by qPCR [41, 42].

#### Independent treatment validation surveys

Independent treatment validation surveys are household-based surveys which can verify routinely reported treatment coverage. The findings are used to carry out any necessary mop-up activities to achieve higher coverage, as well as providing additional information on disaggregated coverage by factors such as age, gender and school attendance. During these surveys, conducted by the Ethiopian Public Health Institute (EPHI), data will be collected on where individuals took the drugs, where they heard about treatment, and reasons for not taking the tablets (including being underage, pregnancy and breastfeeding). These coverage surveys will be conducted within three months of each MDA to identify challenges related to achieving MDA coverage targets and to evaluate reported coverage. The WHO method, adapted from the Expanded Programme on Immunization (EPI) coverage surveys, will be used [43]. Approximately 15 to 20 communities across five districts will be randomly selected to be surveyed each year. Within each selected community, a number of households will be randomly selected, proportional to the size of the community for interview. In each selected household, 2 children (< 16 years-old) and 2 adults (≥  16 years-old) will be randomly selected for an interview.

#### Open defecation free (ODF) verification survey

Eliminating open defecation is a key health outcome of the Geshiyaro Project, given the links to reduced stunting, improved educational and positive health outcomes for children. The Geshiyaro Project has a goal of 40% ODF in Year 1 and 100% from Year 2, to be achieved through CLTS. The approach focuses on community mobilisation to motivate all individuals in a community to understand the health risks associated with open defecation through knowledge, disgust, shame and fear as “triggers” to promote action which ultimately lead to construction and use of household latrines. The FMoH national CLTS protocol used to define and verify ODF [44].

The specific ODF objectives are ambitious and include (i) total elimination of open defecation practices; (ii) 100% coverage of latrine use; (iii) improved personal, household and environmental hygiene; (iv) increased ownership and sustainability of hygiene and sanitation activities; (v) contribution to reduction in sanitation related diseases.

Verification validates the submissions of communities about progress to achieve the stated goals and builds on the key indicators of ODF, i.e. that there is no evidence of open defecation, that households have access to and use latrines. Within each community, verification will occur at various levels (in order):

(i) Verification at the community level by the community administrative team;
(ii) Verification at the district level of each community declaring ODF status;
(iii) Verification by the stakeholder, WVE;
(iv) Independent verification by the Geshiyaro evaluation team. This will include:
  - Review reports: provided by the community, district and WVE records of ODF;
  - Observations: transect walks to observe households in 30% of each community;
  - Interviews: a sample of households will corroborate a visual inspection of the community through interviews with family members; interviews at health centres and schools; community consultations; and key informant semi-structured questionnaires.

#### Formative qualitative research

When trying to develop specific interventions aiming at improving communities’ knowledge, attitudes and practices (KAP), pre-existing capacities must be considered [45]. Qualitative research can bridge identified gaps to enhance the success of the Geshiyaro Project, as health promotion interventions may fail if they are designed without understanding the pre-existing health behaviours of the target population. Furthermore, for interventions focusing on community engagement and those of low socioeconomic status, it is recommended to create a supportive environment for the success and sustainability of strategies [46]. Formative qualitative research will use a mixed method approach to identify different levels of environmental influence on STH and schistosomiasis risk and prevention behaviours. These outcomes will ensure effective BCC, which can be tailored as a comprehensive set of strategies for the control and eventual elimination of schistosome and STH infections.

The formative research will include:

(i) In-depth observations (formative assessment): a walkthrough of common areas, observing the environment for issues of relevance to schistosomiasis and STH transmission cycles. Household observations involving researchers spending periods of time observing daily routines including sanitation and hygiene behaviours, water contact site observations with visits throughout the day and in different seasons to capture different user groups and risk behaviour;
(ii) Household observations: household members including children will be observed for risk exposure behaviours;
(iii) Key-informant interviews: observations will be supplemented by key-informant interviews and focus group discussions (FGD) with community members who are knowledgeable about community norms, perceptions and beliefs, social structure, sanitation and health care services, and drivers of STH/schistosomiasis risk behaviours will be triangulated using key informant interviews with HEWs. Qualitative research will be conducted in Year 1, at mid-point, and endline (Year 5). The endline research will also be performed to measure success in behaviour change strategies and determine lessons learned from the interventions. Appropriate statistical methods will be employed to evaluate changes in both qualitative and quantitative measures from baseline to endline.

The formative research will be carried out by two HEWs from each kebele. In total approximately 15 to 20 kebeles in each arm will be randomly selected to be surveyed each year. This qualitative data triangulated with quantitative WaSH assessment data on hardware coverage to explore core topics that help to understand risk behaviours for acquiring infection and engagement with the interventions (both MDA and WaSH).

#### WaSH assessment survey

There are two major outcomes of the WaSH implementation; total elimination of open defecation through BCC and CLTS, and ultimately the provision of safe water supply and improved latrines.

The WaSH intervention will be assessed through:

(i) Project and service monitoring: school and household level observations of the interventions or activities planned for implementation, evidence of use, cleanliness, photos and GPS coordinates of facilities used both at home and school;
(ii) External evaluation of WaSH will be explored using: (a) the longitudinal parasitological data collection and correlation with WaSH uptake to track programme impact; (b) qualitative assessment of hygiene and sanitation behaviours at baseline, midline and end of the investment to understand whether behaviour has changed in areas that reached transmission interruption; (c) scoring of exposure to infection as determined by WaSH facilities [47]; and (d) formative assessment at any systemic or individual unintended consequences of use of WaSH or behaviour change.

#### Cost-effectiveness survey

In order to assess the scalability of the Geshiyaro Project, the economic costs and benefits of the intervention will be assessed. We will analyse costs as a management tool to understand where to spend resources and where to reduce costs. A combination of prospective and retrospective data will be collected at central, zonal and community levels for analysis [48]. Annual costs will be calculated using the project’s financial data in combination with government estimates, including the estimated value of donated drugs and amortised costs of surveys to assess worm burden. In brief, these calculations will include per diems, training, drug costs, prevalence surveys (mapping and sentinel sites), BCC and sensitisation activities, monitoring and evaluation, and technical support provided both domestically and internationally. Cost data, treatment coverage data and parasite data will be used to determine cost per treatment, cost per additional schistosomiasis and STH control intervention (WaSH), and projections of long-term benefits of investment in elimination strategies.

### Data management

Data from each survey are collected in the field using Samsung J5 smartphones. Questionnaires are coded in SurveyCTO and uploaded from the phones onto the SurveyCTO server. After each year of data collection is complete, the data will be stored on a secure server at EPHI in Addis Ababa, Ethiopia and removed from SurveyCTO. Laboratory parasitological results will be written on paper forms by the laboratory technicians and transferred to SurveyCTO forms at the end of each day.

### Data analysis

Standardised approaches to data cleaning and creation of data sets for analysis will be used. Data will be cleaned in-country based on a series of checks provided by the Monitoring and Evaluation (M&E) team (comprising of staff from EPHI and LCNTDR). “Clean” data will be decided upon by both EPHI and LCNTDR prior to assessment, and issues identified with the data (e.g. missing data, data that appear to be out of a defined range, etc.) to be corrected where possible. Nonsense values (out of known ranges) will be re-coded as missing. Primary data analysis will include the estimation of the prevalence of infection, the intensity of infection and parasite aggregation measures (the negative binomial k value for eggs per gram (epg) counts across age classes) within and between intervention arms. To evaluate the primary outcome, data from baseline, midline and endline will be analysed to determine differences between the arms and to assess what fraction of communities have reached the 2% prevalence threshold for the compilation of positive predictive values for reaching the defined prevalence goal employing data from the end of Phase 2 and the end of Phase 3. To assess overall population-level programmatic impact on the prevalence of STH and schistosomiasis between intervention arms, Generalised Linear Mixed Models (GLMM) will be used in statistical analyses to estimate the differences between arms. Prevalence will be modelled using a binomial (positive and negative) GLMM with logit link function. Unadjusted results will use community and study arm only as covariates. Adjusted results will incorporate pre-determined covariates captured during census, such as urban/rural classification, household sanitation infrastructure, community ODF status, access to adequate water and household socioeconomic status.

Baseline characteristics (captured during census) will be analysed to assess any systematic differences between communities, both within and between intervention arms. This would include number of communities, number of participants, and age, sex, prevalence, and intensity by age classes assessed both by including all individuals and including only egg-positive individuals. Measures of dispersion will be included, for example, interquartile measures of intensity and the negative binomial k value for epg distribution within age groupings. Secondary outcome analysis will include quantifying longitudinal compliance to treatment in conjunction with parasitology data, stratified by age and sex, to determine whether any demographic groups are consistently missing treatment and whether this is associated with infection levels over multiple rounds of MDA. Other secondary analyses will include assessing the change in the intensity of infection and uptake of improved WaSH.

### Process monitoring

The main objective of the Geshiyaro study is to develop a scalable model for STH/ schistosomiasis transmission interruption which can be feasibly rolled out by the health ministries in endemic counties and leads to MDA no longer being required for infection control. The collection and analysis of process monitoring data will ensure intervention facilitators and barriers to implementation are identified. This monitoring of system capacity will be verified through pre-developed tools for preventative chemotherapy such as the NTD Data Quality Assessment Tool (assessing quality of reported NTD data) and the WHO Quality Standards Assessment Tool (assessing the intervention delivery train: including drugs, training, social mobilisation, treatment and reporting).

### Community engagement

Community engagement (sensitisation) is a vital part of all disease intervention studies. It will be performed before each data collection round, specifically covering the activities for that stage of the project. This will comprise of a top-down information dissemination strategy, utilising meetings with community officials prior to the start of data collection. Community official support will be obtained, and the officials will relay the information about the study and how their fingerprints will be used in the study to their respective communities. Secondly, there will be “town-hall” style meetings in the communities presenting the study activities to the population and answering any questions they may have. Sensitisation will cover the aims and outcomes of the project, consent, data capture, MDA and fingerprinting technology. Sensitisation for census, mapping and sentinel sites will be performed by EPHI. Sensitisation for MDA will be performed by FMoH.

## Conclusions

Experience in many settings suggest that chemotherapy has the greatest impact in rapidly reducing the burden and morbidity of helminth infections. However, to accelerate reaching morbidity control and transmission elimination targets, and to sustain improvements, additional measures are ideally needed including WaSH and community public health education. These include a safe water supply, appropriately constructed and maintained sanitation infrastructure that ensures safe disposal of human excreta, the promotion of hygiene such as handwashing plus bathing, and the management of water in the home. Studies have shown that the provision of additional interventions should allow the suppression of the prevalence of STH and schistosomiasis infection to very low levels, achieved through MDA, to be sustained once transmission has deemed to be broken in defined settings such that MDA can cease in these settings [49–51]. The Geshiyaro Project is the first large-scale project, addressing a zone of 1.9 million inhabitants, to provide WaSH facilities combined with MDA within different study arms which permits, in principle, the assessment of the relative benefits of MDA targeted at the whole community, whole community MDA plus WaSH, and MDA targeted at SAC. A cost-effectiveness protocol will be developed to evaluate the cost of both community-wide treatment and WaSH interventions with respect to prevention of heavy and light infections, and therefore the scalability of the Geshiyaro Project to the national level in Ethiopia. If this is successful, it is hoped the experiences in Ethiopia can inform other countries with endemic infection on the design of control policies to achieve the targets defined in the WHO roadmap for 2030 which will be published in 2019. Intensive parasitological assessment using sensitive diagnostics of the effects in each arm of the study has the potential to define an ‘End Game’ for STH and schistosomiasis programmes through provision of multiple interventions. Interrupting transmission of these infections would eliminate the need for long-term repeated MDA, lead to sustained health improvements in children and adults, thereby allowing health systems to focus on other disease control priorities.

## Data Availability

The datasets generated and/or analysed during the current study are available from the corresponding author upon reasonable request.

## Abbreviations

ALB: albendazole
BCC: behavioural change communication
CLTS: community led total sanitation
EPHI: Ethiopian Public Health Institute
FMoH: Federal Ministry of Health
FGD: focus group discussions
HEWs: health extension workers
KAP: knowledge attitude and practice
MDA: mass drug administration
NTDs: neglected tropical diseases
OWNP: One WaSH National Programme
ODF: open defecation free
Pre-SAC: pre-school aged children
POC-CCA: point of care circulating cathodic antigen
PZQ: praziquantel
PCT: preventive chemotherapy treatment
qPCR: quantitative polymerase chain reaction
SAC: school-aged children
STH: soil-transmitted helminths
SNNPR: Southern Nations and Nationalities People’s Region
WaSH: water, sanitation and hygiene
WHO: World Health Organisation
WVE: World Vision Ethiopia
